# Neutrophil Gelatinase-Associated Lipocalin: Biological Aspects and Potential Diagnostic Use in Acute Kidney Injury

**DOI:** 10.3390/jcm14051570

**Published:** 2025-02-26

**Authors:** Grazia Maria Virzì, Niccolò Morisi, Catarina Oliveira Paulo, Anna Clementi, Claudio Ronco, Monica Zanella

**Affiliations:** 1 Department of Nephrology, Dialysis and Transplantation, San Bortolo Hospital, Rodolfi Street 37, 36100 Vicenza, Italy; monica.zanella@aulss8.veneto.it; 2 IRRIV—International Renal Resarch Institute Vicenza, San Bortolo Hospital, Rodolfi Street 37, 36100 Vicenza, Italy; cronco@goldnet.it; 3 Surgical, Medical, Dental and Morphological Sciences Department (CHIMOMO), University of Modena and Reggio Emilia, 41126 Modena, Italy; niccomorisi@gmail.com; 4 Nephrology, Dialysis and Kidney Transplantation Unit, Azienda Ospedaliero-Universitaria di Modena, 41121 Modena, Italy; 5 Intensive Care Department, Hospital Garcia de Orta, 2805-267 Almada, Portugal; catarina.oliveirapaulo@gmail.com; 6 Department of Nephrology and Dialysis, Santa Marta and Santa Venera Hospital, 95024 Acireale, Italy; a.clementi81@virgilio.it

**Keywords:** AKI, NGAL, RRT, lipocalin, biomarker, diagnosis of AKI

## Abstract

Acute kidney injury (AKI) is a syndrome characterized by a rise in creatinine or a decrease in urinary flow, according to the Kidney Disease Improving Global Outcomes (KDIGO) definition. It is diagnosed in 15% of inpatients and 50% of patients in the intensive care unit (ICU), and it is related to increased mortality. As part of a global effort aimed at the elimination of preventable deaths from AKI, there is a growing interest in identifying biomarkers that can be point-of-care and that are not influenced by the variability in patient characteristics in a relevant way. Neutrophil gelatinase-associated lipocalin (NGAL), particularly in its 25 kDa form, which is exclusively released by renal tubules, has emerged as a promising biomarker with potential use in the diagnosis of AKI in the critically ill, including its use in guiding the initiation and/or weaning of renal replacement therapy (RRT). The objective of this review is to summarize the current understanding of NGAL in acute settings, emphasizing biological and genomic insights.

## 1. Introduction

Acute kidney injury (AKI) is a syndrome characterized by a rise in creatinine or a decrease in urinary flow, according to the Kidney Disease Improving Global Outcomes (KDIGO) definition [1]. Recent data indicate that approximately 15% of inpatients and 50% of patients in the intensive care unit (ICU) develop AKI [2,3]. These figures become even more striking when one considers the evidence that links AKI to an increased mortality rate, even in the early stages [4]. Building upon these substantial data sets, the International Society of Nephrology, in collaboration with the European Renal Association, initiated the “0 by 25” initiative with the objective of eliminating preventable deaths from acute kidney injury (AKI) by 2025 in low- and high-income countries [5,6]. In this project, the authors posit that one of the limitations of AKI is the inability to diagnose it rapidly. In order to obtain this, it is necessary to identify AKI biomarkers that are not influenced by the patient’s characteristics [7] or previous measures [8]. Neutrophil gelatinase-associated lipocalin (NGAL), particularly in its 25 kDa form, which is exclusively released by renal tubules [9], has emerged as a promising biomarker with the potential to serve as a point-of-care solution for the early detection of acute kidney injury (AKI) in critically ill patients and as a guide for the initiation and/or weaning of renal replacement therapy (RRT). The objective of this review is to summarize the current understanding of NGAL in acute settings, emphasizing biological and genomic insights, with the aim of exploring its use in clinical practice for both adults and children. For this purpose, we have delved into the biological and genomic aspects to better understand NGAL use in clinical practice.

## 2. Literature Search Tools

A comprehensive search was conducted in PubMed. The references of the selected papers were reviewed to identify additional relevant literature. Case reports, case-series articles, and non-English papers were excluded from the selection. Articles with only abstracts available were also not included. The primary selection criteria were the relevance of the topic, evaluation of the title and abstract, meta-analysis, clinical trials, original research articles, guideline reports, systematic reviews, and recent publications.

## 3. NGAL Discover

Lipocalin 2 (LCN2), alternatively called neutrophil gelatinase-associated lipocalin (NGAL), iron flavin, uterine flavin, and oncogene 24p3, is a soluble protein coming from the superfamily of adipokines [9,10]. In 1989, NGAL was originally discovered by the identification of its messenger RNA (mRNA) in SV40-infected kidney cells in mouse models [11]. In 1993, Kjeldsen [12] et al. isolated NGAL in human neutrophil granules for the first time [12]. Although neutrophils are the principal source of NGAL, it is also present in several human tissues, including tubular cells in the kidney, heart, lung, liver, stomach, colon, epithelial cells, macrophages, dendritic cells, and adipocytes [12,13,14,15,16].

## 4. NGAL: Genomic Organization and Homologous Gene

*NGAL* is located on chromosome 9 (chr9:128,149,071-128,153,453, positive strand). It involves seven exons having at least seven splice variants and encoding for at least five functional transcripts, the most common of which encodes for a 198 amino acid-secreted protein. The *NGAL* mouse gene is the orthologue of human *NGAL* and it is called *lipocalin 2* (*lcn2*). *Lcn2* (NC_000068.8) is encoded by a gene located on mouse chromosome locus 2 (GRCm39, GCF_000001635.27, NC_000068.8 (32274649-32277751). The mouse *Lcn2* gene has six exons and encodes for two functional transcripts [17].

*NGAL* gene is defined as a fast response gene: its expression is modulated by a dose-dependent response induced by various triggers (mostly tissue damage). Its expression is generally up-regulated after toxic insults in a few hours. For these specific characteristics, the speed and the strength of its expression are convenient diagnostic resources for the stratification of patients with specific hazards for evolving tissue damage. These fundamental clinical observations have been replicated in both human populations and animal models [18,19].

## 5. The Protein NGAL: The Lipocalin Family and the Structure

NGAL is defined as a ubiquitous glycoprotein that belongs to the protein superfamily of lipocalin. The lipocalin superfamily is a big cluster of proteins showing remarkable structural and functional variation, both within and between species. Lipocalins are generally small (around 160–180 aminoacidic residues), extracellular proteins characterized by numerous shared molecular recognition capacities. In particular, the binding of small, principally hydrophobic molecules (such as retinol), the binding to defined cell-surface receptors, and the establishment of covalent and non-covalent complexes with other soluble macromolecules are their important features. Although these characteristics are principally classified as transport proteins (binding different ligands and trafficking small molecules to specific appropriate cells), it is now evident that lipocalin family members perform an extensive assortment of dissimilar functions [20]. Some mentioned functions of lipocalins are retinol transport, invertebrate cryptic coloration and olfaction, pheromone carriage, prostaglandin synthesis process, cell growth and metabolism mechanisms, immune response modulation, tissue formation, and action on animal behavior systems [21]. Due to its involvement in various molecular actions and pathways, NGAL has been implicated in a wide range of tissue and cellular processes.

The phylogenetic analysis of the lipocalin protein family found that this family is present in eubacteria and eukaryotic cells (for example: insects and mammals) with a high variety of physiological roles [22,23]. The family shows a great variety at the sequence level (sequence homology approximately 20%); however, most lipocalins are characterized by three typical conserved sequence motifs, whereas a subset of more divergent family members contains only one. Although this is low sequence homology, the crystal structures are highly conserved, particularly the segments of the individual lipocalin proteins known as lipocalin folds. The lipocalin folds are defined by a single eight-stranded continuously hydrogen-bonded antiparallel beta barrel (hydrophobic pocket), which encloses an internal ligand-binding site, essential for their function as transport or carrier proteins of a different selection of ligands [24]. The connection between β-barrel and different protein sheets is represented by seven short loops (L1–L7). Loop L1 forms a lid-like structure to close the ligand binding cavity. The presence of specific amino acids in the lipocalin fold explains the widespread variety of ligands that can be interacted with by lipocalins [20,24]. Additionally, outside of the β-sheet, there is a 3–10 helix at the NH_2_ terminus and an α-helix at the COOH terminus [20]. Human NGAL occurs in three distinguished forms: a 25 kD monomer molecule, a 45 kD disulfide-linked homodimer with Matrix metalloproteinase-9 (MMP-9) form, and a 135 kD heterodimeric form, covalently conjugated with gelatinase. The monomeric form is mainly released by tubular epithelial cells, while dimeric forms are typically secreted by neutrophils that produced also monomeric forms [25,26]. Interestingly, immature neutrophils are characterized by high expression of NGAL mRNA, while mature neutrophils/granulocytes synthesize a high quantity of NGAL protein, stored in specific granules [27]. The main NGAL ligands are siderophores, various groups of small (1 kDa or less) non-peptide iron (Fe3þ)-binding chemicals present in bacteria, fungi, and plants. For this reason, NGAL efforts as a bacteriostatic action appropriating siderophore-bound iron [18,21,28]. In particular, NGAL appears to inhibit the growth and spread of bacterial strains that rely on siderophores to contain their iron necessity [21]. Furthermore, the NGAL iron-binding ability has also been implicated in the delivery of iron to cells and plays a central role in the regulation of iron-sensitive genes that contribute to the epithelial morphogenesis and the mesenchymal–epithelial transition in the formation of the proximal parts of the mammalian nephron [29].

## 6. Baseline State Level

At steady normal state conditions, human biological fluids have extremely low concentrations of NGAL protein. NGAL serum concentration is around 20 ng/mL and it is possibly connected to physiological neutrophils synthesis and minimal liver, spleen, and kidney expression. Renal clearance is a key regulator of NGAL normal level because circulating NGAL undergoes glomerular filtration for its low molecular weight and positive charge [18]. Analogous to serum concentration, urine holds approximately 20 ng/mL of NGAL under physiological conditions. The source and derivation of this protein are not completely defined; in part, it comes from serum NGAL, which bypasses absorption in the proximal tubule (nearly 1/200 molecules). Another option is that NGAL may be derived from neutrophil production or even from bladder epithelia [18].

## 7. NGAL Role in AKI

In a recent review, NGAL was under the spotlight for its involvement in a multitude of diseases, mainly concerning the cardiovascular and renal systems [9]. In a medical setting, it is essential to distinguish between serum NGAL (sNGAL) and urinary NGAL (uNGAL) because they serve as important biomarkers for kidney function, but they provide distinct and complementary information. While both serum and urinary levels of NGAL can indicate kidney injury, they reflect different aspects of kidney health and can aid in the diagnosis, prognosis, and monitoring of patients with acute kidney injury (AKI) or other renal conditions. The former originates from systemic sources, while the latter reflects direct tubular cell injury.

The first study on sNGAL, conducted by Mishra et al. in 2005 with 71 children [30], showed that sNGAL is a sensitive predictor of AKI following cardiac surgery or contrast-induced AKI (CI-AKI). In adults, further studies confirmed sNGAL as a predictor of AKI specifically after cardiac surgery [31,32]. In older patients, sNGAL has demonstrated predictive value for general AKI risk across different clinical settings [33,34]. Additional studies have explored sNGAL’s utility in other AKI contexts, including poisoning [35], sepsis [36], major abdominal surgery [37], and trauma ICU admissions [38]. In the case of uNGAL, Mishra et al. discovered in the same previously mentioned study that it could be a potential tool for detecting AKI following cardiac surgery in children. This observation was corroborated by Sun et al. [39] in a large pediatric population. However, when examining the same outcome in adults with chronic kidney disease (CKD), studies yielded mixed results. The multicenter “PRESERVE trial” by Parikh in 2019 [34], which enrolled 916 adults across 19 centers, demonstrated that uNGAL could predict CI-AKI. Conversely, the single-center “ANTI-CI-AKI study” by Ribitsch et al. [40], which enrolled 607 adults, found that uNGAL failed to detect AKI within 24 h of cardiac surgery. This discrepancy may be due to a delayed rise in uNGAL levels during CI-AKI in patients with CKD, as previously observed [41], though further studies are necessary to confirm this. Similarly, uNGAL was unable to detect AKI in preterm newborns [42]. Finally, the recent research by Brorgi et al. [43] confirmed that in patients with normal or early-stage CKD, uNGAL serves as an important tool for predicting AKI upon ICU admission. Table 1 displays the studies (Table 1). Regrettably, there is considerable variability in the NGAL cut-off values proposed and utilized across various studies, both for serum and urine samples. This discrepancy may be attributed to differences in the assays and methods employed, as well as to the population heterogeneity (i.e., different ethnic origins).

## 8. NGAL and CRRT

Recently, certain studies have investigated the possible role of NGAL during CRRT, exploring its potential as a biomarker for assessing kidney function and predicting patient outcomes in critically ill individuals undergoing renal replacement therapy. Despite the existence of multiple studies on the subject, there is currently no unanimous consensus [45,46,47] on the optimal timing for the commencement and conclusion of Continuous Renal Replacement Therapy (CRRT). The predictive capacity of urine output was found to be adversely affected by the administration of diuretics, which were employed to enhance urine output and facilitate the cessation of CRRT. In light of these assumptions, a preliminary study was conducted in 2018 by Kim et al. [48] to ascertain whether sNGAL could predict the potential for CRRT cessation, but the results were not significant. However, in 2019, Chen et al. [49] demonstrated that sNGAL is well correlated to a successful stop or CRRT at 72 h, but not for septic patients. In the same year, Stande et al. [50] attempted to ascertain whether uNGAL, standardized to urinal creatinine, is associated with CRRT discontinuation. While the initial results were encouraging, with a lower level of uNGAL observed in patients who ceased CRRT effectively, the subsequent multivariate analysis highlighted that there was no statistically significant correlation. On the other hand, in a 2023 study from Japan [51], and in line with previous studies, there was an indication that the uNGAL level at CVVHDF discontinuation reflected renal recovery during CVVHDF. In a pediatric ICU population prospective study of 286 patients, which included patients up to 25 years of age, NGAL was an indicator in a real-time AKI risk stratification-biomarker-directed fluid management protocol of action. When used, results indicated that the median time from admission to Continuous Renal Replacement Therapy (CRRT) initiation was 2 days shorter (*p* < 0.002), and ≥15% pre-CRRT fluid accumulation rate was lower (*p* < 0.02). Length of stay after CRRT discontinuation was also shorter with no statistical significance, and survival rates after ICU discharge in patients who underwent CRRT guided by the protocol, were higher in the comparing group (*p* = 0.001). Healthcare cost reductions were also observed [52]. In Table 2, we summarize these studies (Table 2). Although several studies have addressed the issue, there is still no consensus on the ideal timing for the initiation and termination of CRRT both in the adult and pediatric population.

## 9. Limits for NGAL Utilization

This overview highlights the emerging role of NGAL in acute settings. Unfortunately, there are still limitations and issues that need to be addressed. In particular, there is a lack of standardization in the NGAL values used in clinical practice, as evidenced by various reported studies. This is partly due to the presence of different analytical methods and different laboratory assays and partly due to the heterogeneity of the populations studied. Similarly, there is no consensus on the use of NGAL in the decision of whether to initiate or terminate CRRT. It is also important to note that methods like NGAL are commonly used in high-income countries but they have limited applicability in low-resource countries. Further studies are needed to investigate the usefulness of NGAL in acute settings, focusing on long-term outcomes, such as its prognostic value for mortality or the development and progression of CKD. In addition, the relationship between the pathoanatomic substrate in AKI, such as tubular damage, and NGAL values has not yet been fully explored, and further basic science studies may be needed to explore this point.

## 10. Conclusions

The kidney is a vital organ, comparable to the heart or the brain in terms of its importance. However, in contrast to these organs, the onset of AKI may manifest with subtle symptoms until the point of fatality. In light of this, it is of paramount importance to have an early marker of AKI that can predict the severity of the lesion and the progression of AKI, analogous to troponin for the heart. NGAL, with its rapid response to kidney injury and its tubular secretion, may prove to be a valuable biomarker for early detection of AKI, aiding in early diagnosis, monitoring, and potentially guiding treatment decisions in critically ill patients. In the context of AKI, sNGAL represents a valuable tool for the early identification of disease severity and the necessity of CRRT in acute illness across diverse settings and age groups. The results of studies examining uNGAL in AKI are less encouraging, with a more favorable performance observed in children than in adults. When focusing specifically on CRRT discontinuation, the results do not provide a definitive answer for both sNGAL and uNGAL. However, recent studies offer promising insights.

In conclusion, further research is required to gain a deeper understanding of the role of NGAL in the context of AKI. This includes determining the optimal cut-off values, identifying the most effective timings for measurement, and establishing the necessary number of measurements and intervals to accurately monitor kidney function, guide clinical decision-making, and predict patient outcomes in acute settings. Furthermore, NGAL could play a role in assessing long-term outcomes, such as mortality or the development and progression of chronic kidney disease (CKD). However, additional studies are necessary in this area to better understand its prognostic value and clinical implications.

## Figures and Tables

**Table 1:** Studies on NGAL and AKI.

Country	References	Year	No. and Type of Patients/No.Centers	Biological Fluid	Main Results	Suggested Cut-Off	
						Serum	Urine
Republic of Korea	Ahn, J.Y. et al. [35]	2015	157 adults/1	Serum	NGAL is a good predictor of AKI in poisoning patients.	227 ng/mL on admission	
Australia	Macdonald, S.P. et al. [36]	2017	186 adults/1	Serum	NGAL is associated with illness severity in sepsis and correlates with the inflammatory response.		
Germany	Hunsicker, O. et al. [37]	2017	48 adults/1	Serum	NGAL can be used as an early marker to rule out AKI in patients after major abdominal surgery.	207–210 ng/mL on admission	
Australia	Haase-Fielitz, A. et al. [31]	2018	114 adults/1	Serum	NGAL is a good predictor of AKI after cardiac surgery.	150 ng/mL on ICU admission, 90 ng/mL after 24 h	
Denmark	Walls, A.B. et al. [33]	2021	339 adults/1	Serum	NGAL is associated with AKI in older patients.	139.5 ng/mL on admission	
USA	Mishra, J. et al. [30]	2005	71 children/1	Serum/Urine	NGAL is a sensitive, specific, and highly predictive early biomarker of acute kidney injury after cardiac surgery.	25 ng/mL after 2 h	25 ng/mL after 2 h, 50 ng/mL after 4 h
USA	Parikh, C.R. et al. [34]	2019	916 adults/19	Serum/Urine	Serum NGAL can predict the decline at 90 days after cardiac surgery, urine NGAL can predict the risk of AKI.		
Italy	Quintavalle, C. et al. [32]	2015	253 adults/1	Serum/Urine	NGAL is a good predictor of AKI after cardiac surgery.	179 ng/mL after 6 h	20 ng/mL after 6 h
India	Gupta, B. et al. [38]	2023	140 aduts/1	Serum/Urine	Serum NGAL can predict AKI in trauma ICU patients, urine NGAL is less accurate.	124 ng/mL after 48 h	16 ng/mL after 48 h
USA	Jeffrey C. Sirota et al. [44]	2013	40 adults/2	Urine	NGAL can predict AKI in post-liver transplantation but it needs other urinary markers.		
Denmark	Sellmer, A. et al. [42]	2017	146 neonates/1	Urine	NGAL does not correlate with AKI in very preterm neonates.		
Austria	Ribitsch, W. et al. [40]	2017	607 adults/1	Urine	NGAL fails to detect AKI after 24 hrs of cardiac surgery in CKD patients.		
China	Sun, Q. et al. [39]	2022	172 children/1	Urine	NGAL represents an early and sensitive biomarker for CI-AKI.		36.274 ng/mL after 6 h
Italy	Brogi, E. et al. [43]	2024	23 adults/1	Urine	NGAL at ICU admission could represent a good predictor of AKI.		131.7 ng/mL on admission

**Table 2:** Studies on NGAL and CRRT.

Country	References	Year	No. and Type of Patients/No. of Centers	Biological Fluid	Main Results	Suggested Cut-Off	
						Serum	Urine
USA	Goldstein, S.L. et al. [52]	2023	286 chiltren/	Serum	NGAL helps to manage the fluid administration and CRRT discontinuation in ICU		
Japan	Komaru, Y. et al. [51]	2023	133 adults/7	Urine	NGAL can predict the success of discontinuation of CRRT		186 ng/mL
China	Chen, X. et al. [49]	2019	110 adults/1	Serum	NGAL predicts the successful discontinuation of CRRT in	403 ng/mL	
The Netherland	Stads, S. et al. [50]	2019	92 adults/4	Urine	NGAL is non-significant in multivariate analysis for successful discontinuation		
Republic of Korea	Kim, C.S. et al. [48]	2018	110/1	Serum	NGAL at cessation of CRRT is not associated with successful weaning from CRRT		

## Data Availability

Not applicable.
